# FaceTime for Physicians: Using Real Time Mobile Phone–Based Videoconferencing to Augment Diagnosis and Care in Telemedicine

**Published:** 2011-05-03

**Authors:** David G. Armstrong, Nicholas Giovinco, Joseph L. Mills, Lee C. Rogers

**Affiliations:** ^a^Southern Arizona Limb Salvage Alliance (SALSA), University of Arizona Tueson, AZ; ^b^Amputation Prevention Center, Valley Presbyterian Hospital, Los Angeles, California

## Abstract

**Objective/Background:** Telemedicine has, even in its infancy, had an impact on the provision of healthcare, particularly in rural communities. However, this often relies on an expensive and ponderous infrastructure that reduces the rapid use and spontaneity for consultations. **Methods:** Using postoperative and intraoperative examples, we describe the use of one rapid and widely available technology (iPhone FaceTime, Cupertino, California). **Results:** The device, in allowing “one button connection” similar to making a phone call, reduced the need for preplanning that is generally required for real-time telemedicine consultation. **Conclusions:** The ability to communicate quickly with something that is an afterthought has the potential to alter how we work with our colleagues and patients. Just as with the iPod in music and the laptop in computing, it is not the change in technology, but the change in form factor and ubiquity that alters this landscape.

Medical software and hardware technology is a rapidly progressing and proliferating aspect of patient care.^1^ Often, the newest of such devices are quite expensive to implement and upgrade within an existing patient care facility. These “update costs” are common limiting factors which result in suboptimal delivery of medical service.

As consumer technologies improve and the ubiquity of highly sophisticated and capable devices continue to penetrate into user population, the potential for these costly replacement model could be usurped.^2^ Mobile computing and smartphone handsets, such as the latest iPhone 4 from Apple Inc, demonstrate the ability to mediate the conduction of tele-health service across hundreds or thousands of miles.^3^ The further integration of mobile video conferencing will benefit patient care when successfully negotiating environmental and economic obstacles.^4^

Enabling factors of this kind are fairly unexplored and are potentially vulnerable to several drawbacks.^5^ Compromised quality, security, and versatility may prevent a solution, such as this, from being fully recognized in the ever evolving standard of care for diabetic foot complications.

What is promising about the iPhone 4 and the release of “FaceTime” video calling service is the utilization of open industry standards. This adoption is a decidedly powerful element, which potentiates the previously mentioned challenges to be overcome with a significantly reduced cost of implementation.

In current, the FaceTime augmentation to the phone application supports a number of open standards including H.264 video and advanced audio coding compression and decompression (Codec) standards, session initiating protocol, several network traversal solutions, and real-time multimedia encryption protocols.

## H.264 AND ADVANCED AUDIO CODING CODECS

These multimedia standards offer a number of benefits. They provide a means by which the captured video and audio can be transferred in streaming, real-time to recipient client(s), while maintaining high-quality video. Another added benefit of these codecs is their ability to be utilized on low power and mobile processors, thus limiting battery life and CPU (central processing unit) consumption.

## SESSION INITIATING PROTOCOL FUNCTIONALITY

Streaming multimedia is an inherently challenging process to initiate and maintain. An added difficulty to this process is in the negotiation of a dynamic, party-based feed architecture in which sessions can be commenced and closed in real-time without disruption of host service. The potential for this has been demonstrated in voice over IP service, instant messaging, video conferencing, file transfer, and online gaming.

## NETWORK TRAVERSAL PROTOCOLS

An ever increasing number of hospital and residential environments utilize network Internet access. The nature of this infrastructure is often that of several packet routing intermediates behind firewall security protocols. This arrangement makes for a particularly challenging task for software engineers to design for and, when unsuccessful, results in an inability to perform network operations. Open standard network traversal protocols offer the ability to send and receive data from behind network firewall protection.

## REAL-TIME MULTIMEDIA ENCRYPTION

The consideration of patient privacy and professional confidentiality is of obvious importance. When submitting data through the Internet over a wireless network connection, the potential for compromised security must be accounted for. In addition to compressing multimedia data for rapid delivery, real-time encryption must also be utilized to do so in a safe and secure manner.

Many of these open standards have been developed and maintained by the Internet Engineering Task Force. This organizing body serves as a standards development organization to “improve the usability of the Internet.” This nonprofit entity, and similar working groups, may or may not receive corporate support but provide free use of all patents held to developed property. In the interest of quality, the goal of this sort of volunteer work is to “rough consensus” of opinions with varying levels of expertise. This model, although not perfect, continues to be an important quality of evolving code, which is governed by such and ad-hoc leadership structure.

The potential benefits of this example are numerous. Apple Inc has pledged to commit this application to open standards, which may eventually permit this use and development of this software basis to by third party companies. It is therefore not unreasonable to see this inexpensive platform being extended across multiple personal and hospital devices in future time to come.

## REIMBURSEMENT

Within the United States, some third party payers are reimbursing for “non face-to-face” evaluation and management (E&M) services. In 2008, the American Medical Association added 3 new telephone codes (99441-99443) and one new online code (99444) to report services provided to established patients. There are currently no codes to report physician-to-physician consultation without a direct patient interaction or a new patient consultation by telephone or online.

## LEGAL IMPLICATIONS

In the United States, the practice of medicine is governed by individual states. Physicians are required to hold a medical license in the state where they are providing services, thus imposing a geographical boundary on the practice of medicine. The benefit of the Internet is the elimination of boundaries, but this could pose some issues for physicians not licensed in jurisdictions on both sides of the communication. There is no clear answer if the licensee needs to be licensed in both states and also what constitutes the practice of medicine. It is unlikely that a physician-to-physician interaction would need to be regulated. Doctors attend conferences in localities where they are not licensed and obtain advice for patient care by telephone from colleagues across state lines on a regular basis. Communications across international borders will prove equally complicated legally.

## METHODS

**Figure 1:**
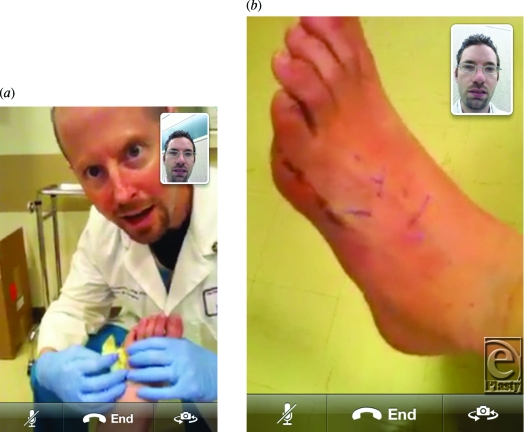
(*a*)–(*b*). In-clinic consultation of postoperative patient, captured from the video stream.

A patient with neuropathy and diabetes underwent a reconstructive foot procedure at the University of Arizona's Southern Arizona Limb Salvage Alliance. At a medical conference, the operating surgeon (D.G.A.) discussed a novel surgical approach with another surgeon (L.C.R.) in passing. The following day, upon returning to clinic, the patient presented for a routine postoperative visit. This spontaneously prompted the “FaceTime” consultation between the two clinicians. Hardware and software limitations of the iPhone yielded only static images to be captured from this video conference event (Figs 1a and 1b). While consent was given for medical photography at the institution, it was also uncomplicated in this case to further isolate and anonymize the anatomy (lower extremity) being evaluated. The novel aspect of this exchange was not the video link between 2 cellular phones, but the fact that it could occur without any preplanning or patient/physician inconvenience.

## RESULTS

**Figure 2:**
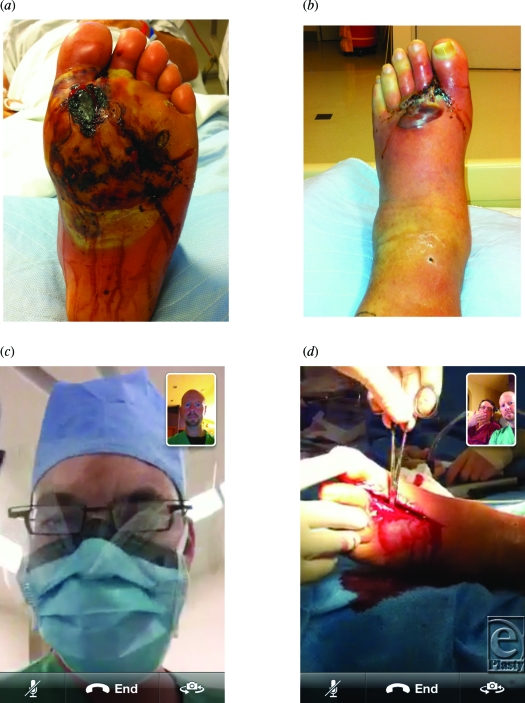
(*a*)–(*d*). Images captured from the intraoperative video consultation of urgent case for surgical planning and staging.

A patient with a limb-threatening infection was taken on an urgent basis to the operating room. With no prior planning except for text messaging, the surgeon consulted with 2 surgical colleagues to discuss incision planning, requirements for resection, and subsequent surgical staging (Figs 2a–2d). The “FaceTime” application was managed by an operating room technician under the direction of the operating surgeon.

## CONCLUSION

We present a potential novel use of seamless phone to phone video technology. We believe that the technology has evolved and disseminated to where spontaneous use of this technology may provide more rapid exchange of information between clinicians. Technologic advances in communication applied to medicine are advancing faster than policy and law. However, these spontaneous interactions will occur naturally and be consumer driven. This, we posit, can only benefit patients.
